# Physics-Based Modelling and Simulation of Multibeam Echosounder Perception for Autonomous Underwater Manipulation

**DOI:** 10.3389/frobt.2021.706646

**Published:** 2021-09-08

**Authors:** Woen-Sug Choi, Derek R. Olson, Duane Davis, Mabel Zhang, Andy Racson, Brian Bingham, Michael McCarrin, Carson Vogt, Jessica Herman

**Affiliations:** ^1^ Naval Postgraduate School, Monterey, CA, United States; ^2^ Open Robotics, Mountain View, CA, United States

**Keywords:** real-time simulation, underwater robotics, multibeam echosounder, point scattering model, gazebo framework

## Abstract

One of the key distinguishing aspects of underwater manipulation tasks is the perception challenges of the ocean environment, including turbidity, backscatter, and lighting effects. Consequently, underwater perception often relies on sonar-based measurements to estimate the vehicle’s state and surroundings, either standalone or in concert with other sensing modalities, to support the perception necessary to plan and control manipulation tasks. Simulation of the multibeam echosounder, while not a substitute for in-water testing, is a critical capability for developing manipulation strategies in the complex and variable ocean environment. Although several approaches exist in the literature to simulate synthetic sonar images, the methods in the robotics community typically use image processing and video rendering software to comply with real-time execution requirements. In addition to a lack of physics-based interaction model between sound and the scene of interest, several basic properties are absent in these rendered sonar images–notably the coherent imaging system and coherent speckle that cause distortion of the object geometry in the sonar image. To address this deficiency, we present a physics-based multibeam echosounder simulation method to capture these fundamental aspects of sonar perception. A point-based scattering model is implemented to calculate the acoustic interaction between the target and the environment. This is a simplified representation of target scattering but can produce realistic coherent image speckle and the correct point spread function. The results demonstrate that this multibeam echosounder simulator generates qualitatively realistic images with high efficiency to provide the sonar image and the physical time series signal data. This synthetic sonar data is a key enabler for developing, testing, and evaluating autonomous underwater manipulation strategies that use sonar as a component of perception.

## 1 Introduction

Simulation of robotic manipulation systems has proven its usability and effectiveness for designing, testing, and evaluating its capability (Cook et al., 2014). Especially for underwater environments, operational demands make testing the physical hardware costly and risky. It is critical to develop manipulation strategies in complex and variable ocean environments. Therefore, simulation capability to test rapidly, and at low cost is critical to test new designs and system control strategies. While simulations cannot replace in-water testing, the cost of development of manipulator systems can be reduced using accurate simulators.

Manipulation has made much progress in recent years, notably from images and point clouds. Underwater manipulation has been carried out using multi-sensor suites (Sanz et al., 2013; Cieslak et al., 2015), and underwater sensors have been developed (Palomer et al., 2018) to leverage perception methods developed for on-land sensor data. Additionally, because there is a diversity of underwater vehicle and manipulator types and classifications, without an authentic simulation capability, matching perception and control solutions to specific scenarios relies exclusively on field testing. Physics-based simulation enables early evaluation of novel approaches to specific challenges, e.g., model free approaches to under-actuated scenarios (Tutsoy and Barkana, 2021).

One of the key distinguishing aspects of underwater manipulation tasks is the perception challenges of the ocean environment, specifically turbidity, backscatter, and lighting effects. High-fidelity simulations of sonar-based perception are essential for bathymetric maps or obstacle avoidance as well as to support manipulation planning and control to enable accurate feedback (Manhães et al., 2016). Autonomous manipulation poses higher demands on perceptual accuracy, on the scale of centimeters, than larger scale operations and fidelity to conduct intervention tasks requiring physical contacts in unstructured environments and without continuous human supervisions.

Sonar-based perception is particularly challenging due to the slow propagation speed of sound compared to electromagnetic waves, and the large bandwidth to center frequency ratios of sonar applications. This challenge leads to a large amount of data produced by acoustic systems, and correspondingly a large computational load to simulate high-fidelity synthetic data, particularly for real-time simulations.

### 1.1 Previous Work

Several approaches have been developed in the literature for the open source Gazebo robot simulator (Koenig and Howard, 2004) which has emerged as a standard environment for the robotics community. In DeMarco et al. (2015) a Gazebo sonar sensor model is developed using ray tracing. The Gazebo ray-tracing functionality generates a 3D point cloud which is transformed into a sonar image. On inspection, the acoustic properties were either hard-coded or not considered and did not include speckle noise or time-angle ambiguities. The latter two aspects are standard acoustic pulse echo imaging characteristics that are discussed later. In Cerqueira et al. (2017, 2020), a GPU-based sonar simulator is developed using rasterization. They model two types of sonar: a mechanically scanned imaging sonar (MSIS) and a forward-looking sonar (FLS). The acoustic features provided in their model exploits precomputed acoustic parameters to convert the image into a synthetic sonar image using geometric information provided by the rasterized image. The acoustic precomputed parameters to render the camera image into a sonar image includes three components: pulse distance, echo intensity, and field-of-view.

### 1.2 Shortcomings of Previous Work

Previous methods in the literature are based on image processing and video rendering of the simulated rays intersecting a target to convert into a sonar image pixel-wise and image-based manner (DeMarco et al., 2015; Cerqueira et al., 2017; Cerqueira et al., 2020). Overall, a grid is formed for the acoustic image data, and any rays that intersect a given pixel are added to it incoherently. These approaches have several significant shortcomings, including a lack of physically accurate interaction model between sound and the object of interest, neglect of time and angle ambiguities that are present in any pulse-echo imaging system, and a lack of speckle noise that is present in a coherent imaging system. The time and angle ambiguity is a function of the point spread function of the coherent imaging system [i.e., side lobes due to matched filtering and beamforming, van Trees (1971)]. Speckle is the granular appearance of an image that is due to many interfering scatterers that are smaller than the resolution limit of the imaging system (Goodman, 2015) that cause distortion of the object geometry in the sonar image.

For any task that requires geometric information obtained from sonar data (such as autonomous manipulators), these basic acoustic properties must be accounted for in the simulation. In addition to the limited fidelity of the previous image-based approaches, they generate only post-processed imagery of the underlying acoustic measurements to qualitatively approximate the interface provided to a human operator through a sonar viewing application. Autonomous manipulation, perception, planning and control rely upon access to the sensor-level measurements that are independent of the viewer used to render the measurements into imagery.

This study presents a beam-level time series sonar simulation method to provide a range versus intensity data that underwater vehicle manipulator systems can exploit. A point-based scattering model is implemented, following Brown et al. (2017) to calculate the interaction between sound waves and the target and environment. This is a simplified representation of target scattering, but is able to produce realistic coherent image speckle, and the correct point spread function. Also, with the underlying assumption of each beam consisting of a sum of independent scatterers on the target, this model can be easily parallelized using a GPU (Graphics Processing Unit) to provide a practical refresh rate. The refresh rate is the rate per unit time at which datasets are produced by the sensor simulation components, which is typically user-defined but limited by computational capabilities of the simulating hardware. This model was implemented within the Gazebo framework to provide both real-time sensor-level beam intensity data and scene rendering.

The application results based on proposed methods demonstrate that this multibeam echosounder simulator generates qualitatively realistic images with high efficiency to provide the sonar image and the physical time series signal data in real time with sufficient refresh rate showing its effectiveness and usability. This sonar data is a key enabler for developing, testing, and evaluating autonomous underwater manipulation strategies that use sonar as a perception component.

### 1.3 Contribution

The contributions of the methods in this article are to virtually produce sonar perception data with appropriate fidelity and within sufficient refresh rates. Collectively, the methods can provide physical time series signal data to improve the simulation infrastructure that underwater manipulation strategies and systems can exploit. Individually, our contributions are as follows:

• A simplified physics-based forward looking echosounder with a point-based scattering model within Gazebo framework to support underwater vehicle simulations
• High fidelity acoustics simulation including multibeam, scattering, noise, and target-wise reflectivity to increase the fidelity of current capabilities and generate sensor-level beam intensity measurements suitable for exercising autonomous manipulation perception, planning and control
• GPU parallelization for real time sonar simulation data processing

## 2 Methods

The model is based on a point scattering model of the echo level using the ray-based spatial discretization of the model facets as scatterers corrected with beam pattern appropriate for a line array. We first present the geometric information provided by Gazebo for each beam, then detail the calculations need to produce an intensity time series for each beam.

### 2.1 Single Beam Sonar Model

**FIGURE 1:**
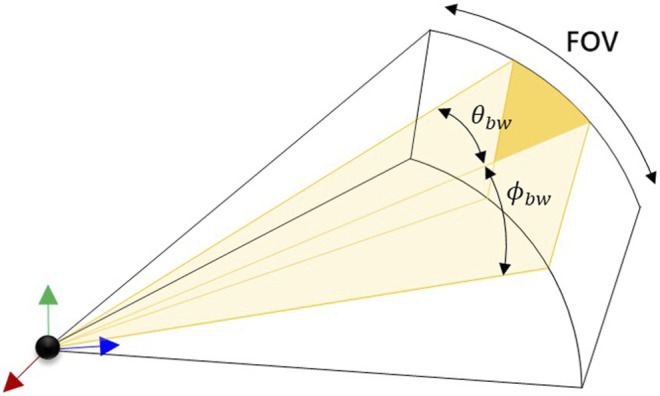
A single sonar beam with its azimuth and elevation beam widths.

**FIGURE 2:**
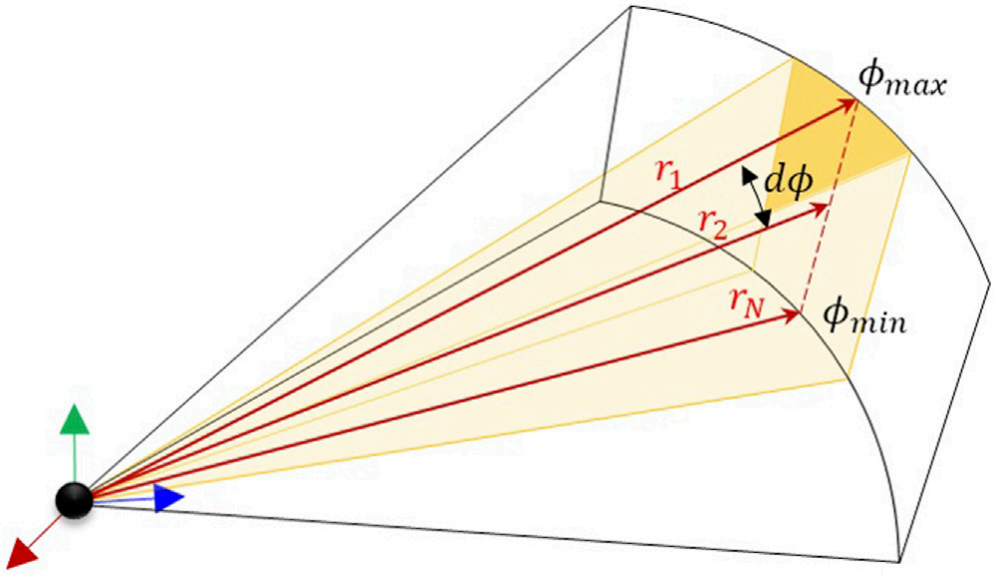
Set of rays forming a single sonar beam.

**FIGURE 3:**
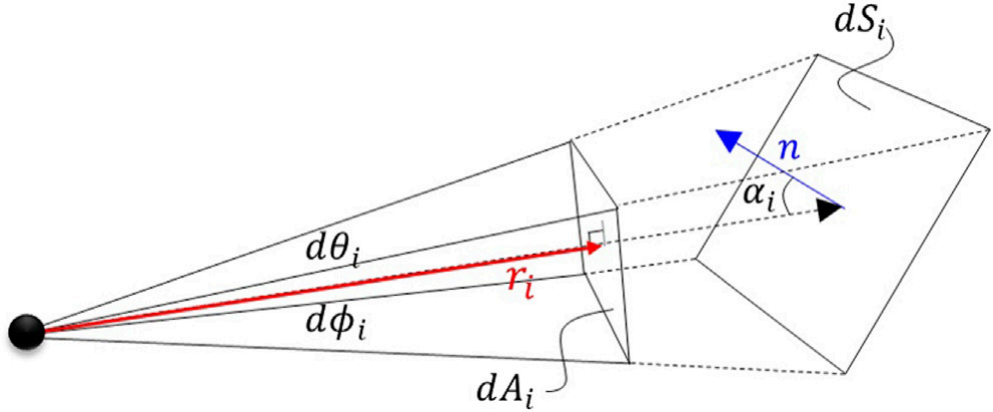
Projected ray surface area and surface area patch of the visual object.

A single sonar beam within the field of view (FOV) of the sensor is shown in Figure 1. An ideal sonar beam pattern is a unit gain within the orange shaded region, and zero response outside of it. In reality, the beam response exists over all angles, although the major contribution is within the beam width of that particular beam. Here, a beam is modeled using discrete rays. The individual rays are indexed as *i* = {1, 2, … *N*} for *N* rays and beams are indexed as *j* = {1, 2, … *N*_*B*_} for *N*_*B*_ beams. Individual rays correspond to each scatterer on the target mesh. The following information is generated for each ray within an individual beam:

• The range *r*_*i*_ as the distance from the origin of the sonar reference frame to the first intersection between the ray and a target in the field of view. The azimuth of the ray is fixed in the sensor frame as *θ*_*i*_ and the elevation angle of the ray as *ϕ*_*i*_ (Figure 2).

• The incident angle *α*_*i*_ as the difference between the ray vector, **z**, and the normal vector, **n** of the target surface at the location of intersection between the ray vector and the target surface (Figure 3).

• The reflectivity of the target intersected by the *i*-th ray, *μ*_*i*_, which is a property of the target object model (shape, size, and material), and the sonar frequency and bandwidth.

This scene information is provide by the Gazebo 3D simulation framework at each execution cycle (typically 1 kHz). The scene information is then used as inputs to the sonar model described below to generate an acoustic time series. The range and the normal vector of the intersected target elements in the environment are obtained from the depth buffer interface to the Gazebo 3D rendering pipeline. The locations in the depth buffer are then correlated with the visual scene rendering to deduce the target reflectivity. The methods to calculate the time series are detailed below.

### 2.2 Ray-Based Beam Model

We define a ray as a vector, **z**_*i*_, from the sensor frame origin to the first intersection with a visual object within the scene. We calculate the incidence angle (angle between the surface normal and the intersecting ray) as

αi=180°−cos−1(z^i⋅n^i) (1)

where ${\hat{z}}_{i}$ and ${\hat{n}}_{i}$ are the ray and normal direction unit vectors respectively.

The projected ray surface area, *dA*, is the area projected onto the visual object by the individual ray as shown in Figure 3. If the changes in both *dθ*_*i*_ and *dϕ*_*i*_ angles for each ray are assumed to be infinitesimally small, then the projected area ray scene can be calculated by

$$dA_{i}=r_{i}^{2}d\theta_{i}d\phi_{i}$$ (2)

Using the ray area projected onto the surface of the model element, *dS*_*i*_ is a function of the incident angle, calculated as

$$dS_{i}=\frac{dA}{cos\left(\alpha_{i}\right)}$$ (3)

and expressed as

$$dS_{i}=\frac{r_{i}^{2}d\theta_{i}d\phi_{i}}{cos\left(\alpha_{i}\right)}$$ (4)

This equation relates the geometry of the ray to the surface area of the element that intersects the ray.

The target strength of the model element, which represents the ratio of scattered to incident intensity, is given by

$${TS}_{i}=10\log\frac{I_{sca}}{I_{inc}}$$ (5)

where *I*_*sca*_ is the intensity scattered by the target measured at 1 m distance, and *I*_*inc*_ is the incident intensity on the target. Models for target strength are typically complex, and depend on the shape and geometry of the target (Williams et al., 2010; España et al., 2014; Kargl et al., 2015). Since the goal of this work is to provide a realistic simulation of the geometry of a target, but not its amplitude, we use the empirical Lambert’s law for scattering. Using Lambert’s empirical model, the ratio of scattered to incident intensity is

$$\frac{I_{ri}}{I_{Ii}}=\mu \left(cos^{2}\left(\alpha_{i}\right)\right)dS_{i}$$ (6)

where *μ* is a parameter that controls the overall reflectivity to the scattered field. This model is commonly used when the surface is very rough compared to the wavelength and represents a perfectly diffuse scattered field, and often provides a good fit to seafloor reverberation measurements (Lambert, 1760; Prior, 2005; Holland, 2005; Olson et al., 2016; Boehme et al., 1984). This approximation is reasonable in this case, since the sensors use a very high frequency center frequency with a wavelength on the order of O (1 mm). If a target’s surface is even slightly rough, then the scattered field will be diffuse. This can occur if the target is manufactured with some surface roughness, or if it has been subject to biofouling (Lewis, 1998), which is very common.

Substituting for *dS*_*i*_, the intensity ratio becomes

$$\frac{I_{rs}}{I_{Ii}}=\mu \left(cos\left(\alpha_{i}\right)\right)r_{i}^{2}\;d\theta_{i}d\phi_{i}$$ (7)

this expression is used below to calculate the amplitude of the point scatterer amplitude.

### 2.3 Ray-Based Point Scattering Model

Synthetic time series are simulated using the point-based scattering model of Brown et al. (2017). Although this model was developed for the seafloor, it can be used to characterize the incoherent field scattered by a target. It can also be adapted to generate a coherent component if that is desired. The model generates a spatially coherent time series that is useful in simulating narrowband sonar applications such as multibeam echosounder (MBES) systems. The model uses discrete scatterers distributed over a surface defined by a discretized model mesh. These scatterers are representative of the number of surfaces a ray intersects based on the object’s surface mesh.

Here, we define each ray intersected surface mesh element as a scatterer; the number of rays is equivalent to the number of scatterers. This approach is valid so long as the ray intersecting surface mesh is discretized with an average ray spacing smaller than the resolved area of the system. If this criterion is not satisfied, then the number of rays in a beam should be increased before use in this ray-based scattering model.

The overall spectrum of the signal received from each beam, *P*_*j*_(*f*), is computed as

$$P_{j}\left(f\right)=S\left(f\right)\overset{N}{\underset{n=1}{\sum }}\frac{a_{i}D\left(\theta_{i},\phi_{i}\right)e^{i\left(2\tilde{k}r_{i}\right)}}{{\left(r_{i}\right)}^{2}}$$ (8)

where *S*(*f*) is the transmitted spectrum of the source, *N* is the number of scatters, *a*_*n*_ is the complex scatterer amplitude, *f* is the acoustic frequency in Hertz and $\tilde{k}=k_{w}+ik_{w}^{\prime }$ is the complex, wave number with real part *k*_*w*_ = 2*πf*/*c*, and imaginary part *k*_*w*_′. *c* is the medium sound speed, in this case seawater. *P*_*j*_(*f*) is computed as a combination of the physical model for echo level and a complex random scale factor for speckle noise resolved in the frequency domain. Attenuation is included as the imaginary part of the wavenumber, and can be related to other conventions for attenuation (e.g., dB per m) using formulae in Ch. 8 of (Jackson and Richardson, 2007). The acoustic frequency is *f* in hertz. The subscript *j* represents the index of the beams computed. A time series for each nominal beam angle is produced. The directivity pattern of beam *j* is denoted by *D* (*θ*, *ϕ*) and is a function of the azimuthal angle, *θ*, and elevation angle, *ϕ* between the sensor and the scatterer. The directivity function to model the beam directivity pattern is discussed in Section 2.4. This equation is the most complex component of the model to calculate.

The source spectrum is a user defined input and remains constant for each ray and is modeled in the frequency domain by *S*(*f*). In this model, we use a Gaussian model with a center frequency, *f*_*c*_ and bandwidth, *b*, parameters controlling the location and width of the spectrum respectively, as in

$$S\left(f\right)=S_{0}e^{-{\left(f-f_{c}\right)}^{2}b^{2}\pi^{2}}$$ (9)

The Gaussian form here is simple, but in any realistic simulation, the spectrum of the wave transmitted by the sonar system, and realistic filtering by the sonar receiving subsystem should be used. The source parameter by *S*_0_ and has units of Pa⋅m. The decibel version of *S*_0_ is the source level (Urick, 2013).

To obtain synthetic time series, acoustic frequency is discretized into linearly spaced vector from *f*_min_ to *f*_max_ and centered on the center frequency, *f*_*c*_. The full width of the transmit spectrum is the bandwidth, *b*, a user provided input based on the sonar specifications. As an example, the bandwidth for the BlueView P900 Series FLS is 2.95 kHz ^1^ (Teledyne BlueView Inc, 2015). The *m*-th member of the frequency vector, with *m* ∈ [1, *M*].

$$\begin{array}{ll} f_{m} & =m\Delta f+f_{min} \end{array}$$ (10)

$$\begin{array}{ll} f_{min} & =f_{c}-\frac{b}{2} \end{array}$$ (11)

$$\begin{array}{ll} \Delta f & =\frac{1}{T} \end{array}$$ (12)

$$\begin{array}{ll} M & =bT \end{array}$$ (13)

where Δ*f* is the frequency spacing, *T* is the desired temporal duration of the signal. The number of frequencies is *M*. The acoustic frequency vector can be mapped to the wavenumnber via *k*_*m*_ = 2*πf*_*m*_/*c*. Once the frequency-domain response is calculated, then the time-domain response can be computed using an inverse Fourier transform, typically implemented using the fast Fourier transform algorithm.

Spherical spreading is an appropriate assumption for modeling acoustic propagation at short ranges. The two-way transmission loss for incoherent scattering in *P*_*j*_(*f*) is captured in the denominator, $2r_{i}^{2}$, where 2*r*_*i*_ is the two-way distance (from source to scatterer, and back to the receiver).

The scatter amplitude, *a*_*i*_, is calculated for each ray, and is related to the target strength discussed in Eq. 7. It is calculated using,

$$a_{i}=\frac{\xi_{xi}+i\xi_{yi}}{\sqrt{2}}\sqrt{\mu_{i}cos^{2}\left(\alpha_{i}\right)r_{i}^{2}d\theta_{i}d\phi_{i}}$$ (14)

The variables *ξ*_*xi*_ and *ξ*_*yi*_ are draws from independent Gaussian random variables. Although the random variable, *ξ*_*i*_, is indexed by *i* to represent the ray index, the real random variable and the complex random variable must both be generated and different from each other, hence the *x* and *y* notation. Overall, the random variables are representative of Gaussian noise and for our purposes, satisfies the speckle noise requirement (Brown et al., 2017). If the target is very smooth, then the scattered field will contain a coherent component, which can be represented by an additive constant term to *a*_*i*_. The summation of complex Gaussian noise and a coherent field would result in Rician statistics (Rice, 1944). The variables under the square root represent the target strength of an incident ray on an object.

### 2.4 Directivity Pattern Model

A realistic acoustic image should show the side lobes of the beam pattern of a single point target. In some applications, it is advantageous to simulate the time series of each element of the receive array, and perform beamforming. This method is accurate, as it reproduces the signal processing of the sonar sensor exactly, but may require proprietary signal processing algorithms used in the system. However, simulating channel-level acoustic measurement, prior to beamforming, is computationally intractable for real time execution, at least using current computing hardware. To mitigate this inefficiency, we simulate each beam assuming that the horizontal beam pattern is a uniform, ideal beam pattern within the beamwidth. The time series are generated for a fan of beams whose directions correspond to the beamformed directions for the particular multibeam echosounder. The ideal beams are then corrected by performing a weighted sum of the beams, detailed below. This method does not perfectly reproduce the time series generated from the more accurate method, but should be sufficient so long as there are no extremely strong scatterers outside the fan of ideal beams to be simulated.

**FIGURE 4:**
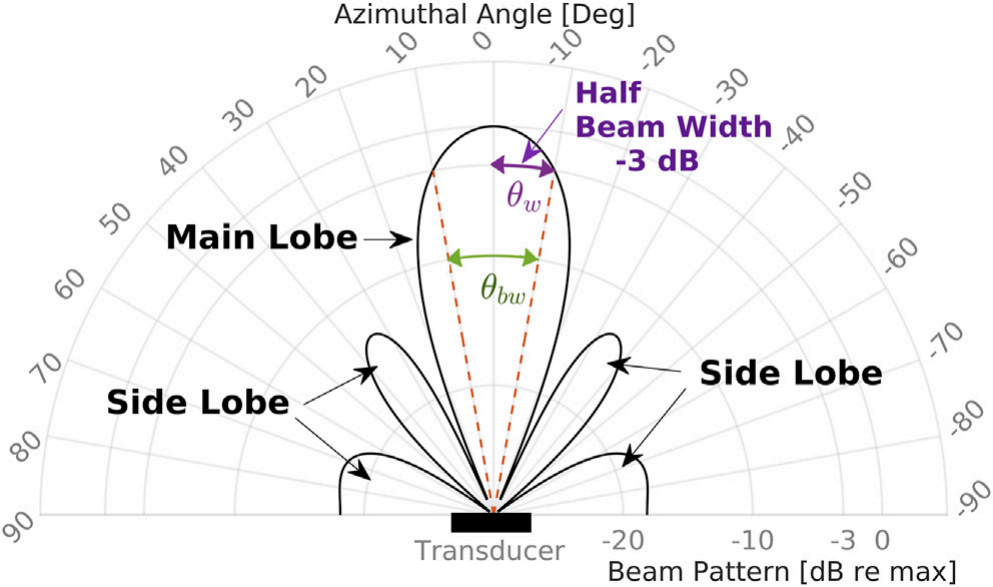
Beam pattern schematics of half power beamwidth.

The beam pattern of an array is defined in polar coordinates where the acoustic intensity is the distance along the radial axis and the angle is relative to the transducer axis. The beam pattern is visualized as one main lobe in the center with smaller side lobes radiating away from the main axis (Figure 4).

By inspection, the highest return will be along the main axis, and the response decreases off-axis. Local maxima appear away from the main axis and are referred to as side lobes. Any acoustic image or measurement will be subject to side lobes, which introduces some ambiguity into finding a target’s direction in sonar data. This ambiguity is present when using any coherent imaging system that uses multiple receivers (van Trees, 1971), similar to the effect of spectral leakage in spectrum estimation (Harris, 1978). The side lobe level is the decibel of ratio of the peak of the first side lobe to the main lobe peak, and is one way of quantifying the corruption of an acoustic image due to side lobes. Therefore, the echo intensity of a patch on a target depends on the size and position within a given beam. The beamwidth, *θ*_*bw*_, is marked at −3 dB on the main lobe. We define half the beam widths

$$\theta_{w}=\frac{\theta_{bw}}{2}$$ (15)

as the one-way angular distance between the main response axis and the half power point.

The effect of side lobes can be simulated by performing a correction whereby each beam is a weighted sum of all the other beams. The weight is the directivity pattern of the array with the main response axis steered in a particular direction, as in

$$p_{j}\left(t\right)=\frac{\overset{N_{B}}{\underset{i=1}{\sum }}p_{i}\left(t\right)w_{i,j}}{\sqrt{\overset{N_{B}}{\underset{i=1}{\sum }}|w_{i,j}{|}^{2}}}$$ (16)

where *w*_*i*,*j*_ is the weight for the *i*-th beam steered in the *j*-th direction. So long as the fan of beams is sampled at less than the 3 dB beam width (ideally at least half the beam width), this summation reproduces the correct effect of side lobes in the resulting time series. The form of the sum used here preserves the mean square value between the corrected pressure, and the initial simulated pressure.

The weights are the values of the beam pattern sampled at specific angles. In this work, we use the directivity pattern corresponding to a uniform linear array,

$$w_{i,j}=B\left(\theta_{i}-\theta_{j}\right)$$ (17)

where *θ*_*i*_ is the horizontal angle corresponding to the *i*-th and *j*-th beam respectively. The beam pattern, *B* is defined in the following paragraphs.

The beam pattern, *B*(*θ*), expresses the pressure ratio of the response of the array at an angle *θ*, relative to the main axis. For a continuous line array of length *L*, radiating energy at a wavelength *λ* = 2*π*/*k*_*w*_, the beam pattern is that of a uniform aperture function. The radiated pressure is modeled as a normalized *sinc* function,

$$\begin{array}{l} B\left(Lu\right)=sinc\left(Lu\right) \end{array}$$ (18)

$$\begin{array}{ll} & =\left\{\begin{array}{cc} 1,\; & for\;Lu=0\\ \frac{\sin\left(\pi Lu\right)}{\pi Lu},\; & otherwise \end{array}\right., \end{array}$$

where *u* is the electrical angle

$$u=\frac{\sin\left(\theta \right)}{\lambda }$$ (19)

The half intensity point, *θ*_*w*_, can be solved for by setting

$$|B\left(\theta_{w}\right){|}^{2}={\left|\frac{\sin\left(\pi \frac{L}{\lambda }\sin\left(\theta_{w}\right)\right)}{\pi \frac{L}{\lambda }\sin\left(\theta_{w}\right)}\right|}^{2}=\frac{1}{2}$$ (20)

For high frequency sonar, we can assume *L* ≫ *λ*, and use the small-argument approximation to the inner sin function, and *θ*_*w*_ becomes

$$\frac{L}{\lambda }\approx \frac{0.884}{\theta_{bw}}=\frac{0.442}{\theta_{w}}$$ (21)

The final beam pattern is

$$B\left(\theta \right)=\frac{\sin\left(\pi \frac{0.884}{\theta_{bw}}\sin\left(\theta \right)\right)}{\pi \frac{0.884}{\theta_{bw}}sin\left(\theta \right)}$$ (22)

here, beam pattern, *B*, can be either positive or negative, depending on the beam angle *θ*. When squared, it captures the intensity version of the beam pattern.

The overall algorithm to calculate the sonar model is shown in Algorithm 1. To generate a single pair of range and intensity values for each beams (*r*_*j*_, *I*_*j*_) for *j*-th beam, using ray-based beam model (Eq. 8), the beam pattern in Eq. 22 is applied to each sampling rays. Each sample is a ray at *ϕ*_*i*_ in the range [−*ϕ*_*w*_, *ϕ*_*w*_] with the associated range and intensity pair (*r*_*i*_, *I*_*i*_). Here, *ϕ*_*w*_ is equivalent to *θ*_*w*_ for elevation angles. The set of ordered pairs from all rays is *i* = {1, … , *N*} used to construct a single pair of (*r*_*j*_, *I*_*j*_) for a beam. Thereafter, for interference between beams, Eq. 16 is applied with the set of pairs from all beams *j* = {1, … , *N*_*B*_} as a corrector.

Algorithm 1 Multibeam echosounder calculation algorithm.

**Table:** 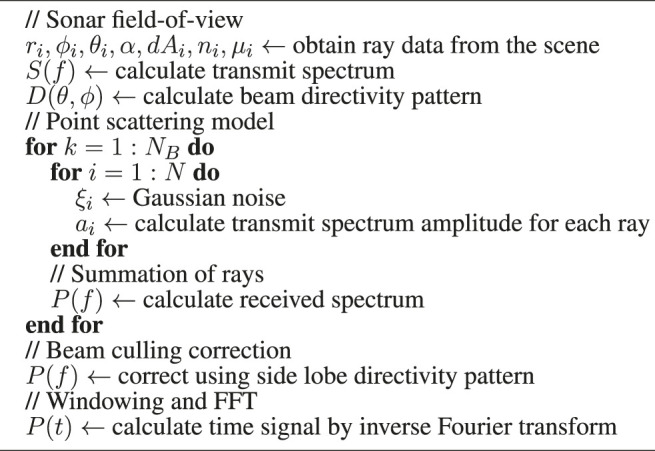
.

### 2.5 Gazebo and Robot Operating System Implementation

**FIGURE 5:**
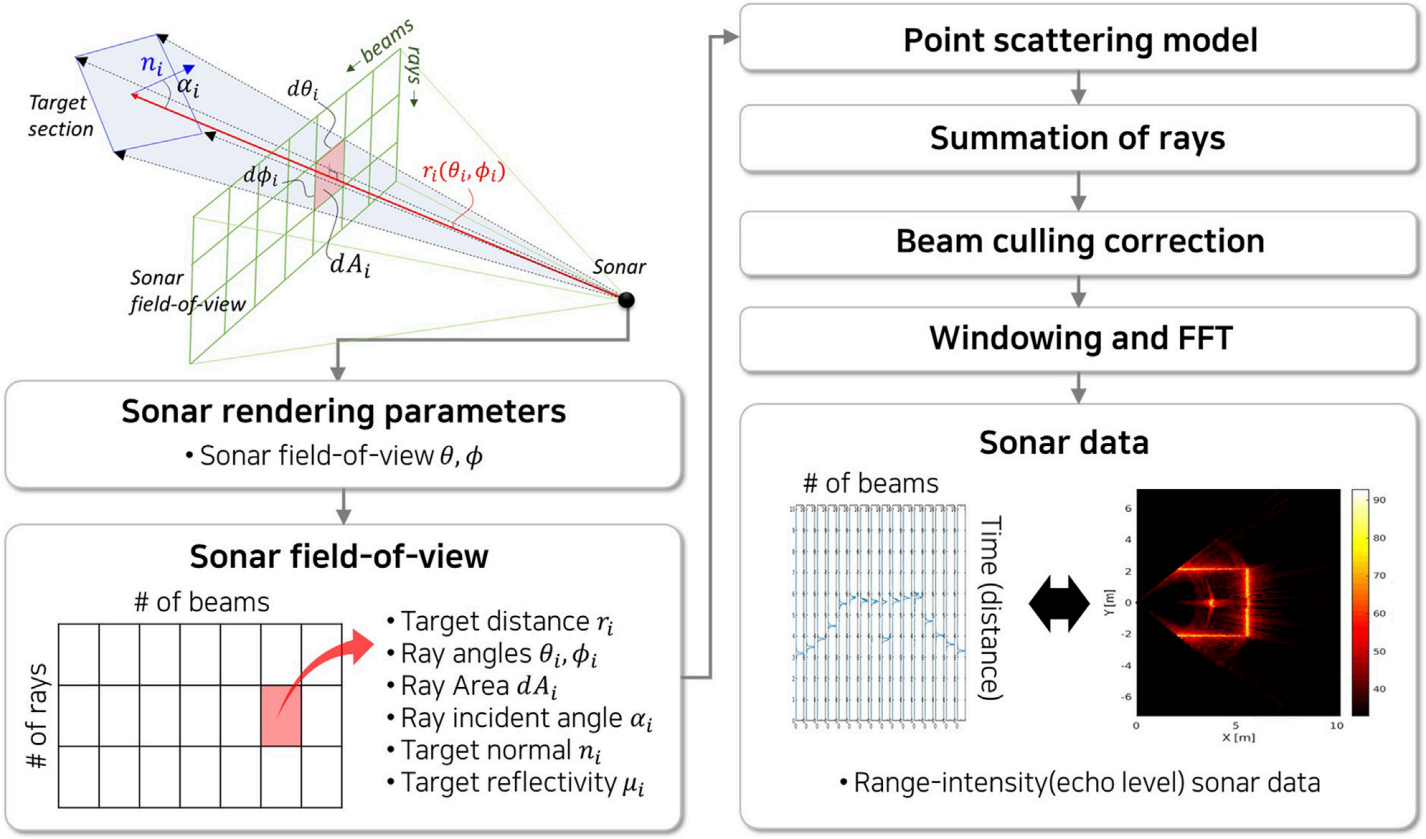
Overall procedures of the imaging sonar simulation process: (i) a Gazebo camera plugin obtain the underwater scene; (ii) two dimensional set of sonar field-of-view data captured in the rendering scene; (iii) point scattering model is calculated for each ray data (iv) summation of rays to each beam; (v) beam pattern effect is calculated for beams; (vi) and the windowing and fast-fourier transform (FFT) is performed to produce range-intensity sonar data for each beam.

The Gazebo simulator and Robot Operating System (ROS) have become *de facto* standards for robotic simulations. Gazebo simulates various perception sensors using modular plugins in the environment. The sonar model is implemented in Gazebo as shown in Figure 5 as released as open source^2^. Based on Gazebo’s camera plugin, the sonar field-of-view data is rendered from the scene using azimuth and elevation field-of-view angles of the actual sonar to generate three dimensional point clouds of the target objects. The point cloud resolutions are set to match with the number of beams in the sonar in azimuth angles and multiple rays in the elevation angles. Each ray in two dimensional sonar field-of-view data consists of target distance, ray angles, ray area, ray incident angle to the target, target normal, and prescribed reflectivity of the target.

Using a GPU with the NVIDIA CUDA library^3^ (Vingelmann and Fitzek, 2020), each ray is computed in parallel using Eq. 8 to generate signal spectrum. Here, the complex scatter amplitudes are calculated for each ray accordingly using the sonar field-of-view data. Thereafter, ray spectra are summed to calculate the beam signal spectrum.

In order to apply beam pattern effect, the weights in Eq. 16 are computed. To further reduce the computation time, the calculation is also performed as matrix multiplication using the GPU with the weights (Eq. 17) pre-calculated when sonar rendering parameters are fixed. Finally, matrix multiplication for Gaussian windowing and Fourier transform are also performed using GPU to generate range-intensity signal data for each beam.

The final signal data of the sonar simulation is produced in a custom ROS message format^4^ by UW-APL (2021) which is a community-driven standardized ROS message for hydrographic applications. The message format is designed to match that of the physical sonar applications. The final sonar signal data can be mapped onto polar coordinates to generate a sonar image.

## 3 Real-Time Multibeam Echosounder Simulation

**FIGURE 6:**
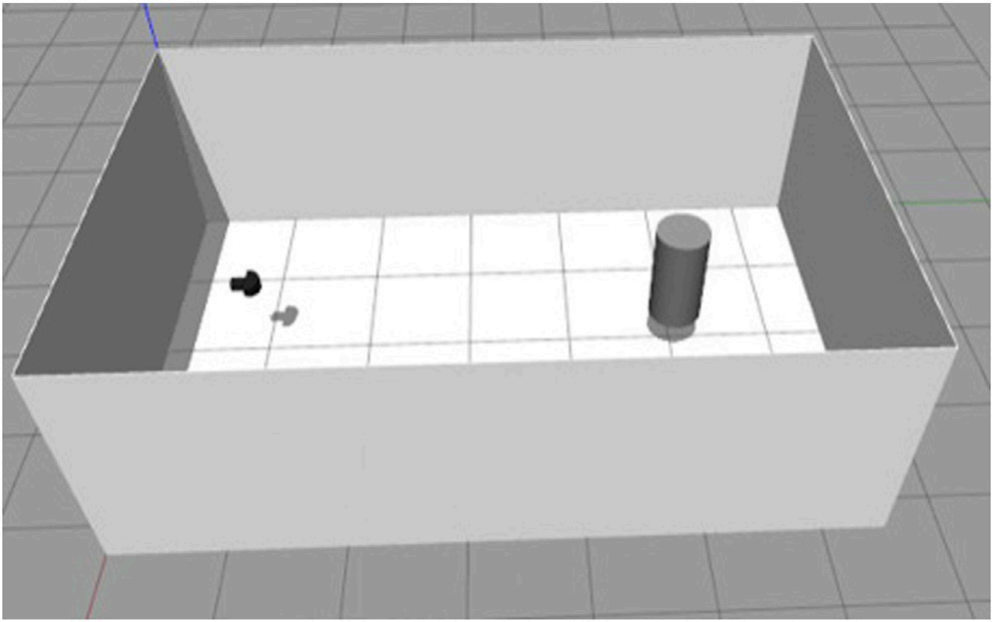
A multibeam echosounder set in side the square sonartank with a cylinder target.

To evaluate our simulator, the sonar model with the developed plugin is set inside a square sonar tank with a cylinder as a target as shown in Figure 6. The distance between the 0.4 m diameter cylinder and the sonar is 4 m and the wall on the opposite side of the tank is 5.5 m. The sonar configurations are summarized in Table 1.

**TABLE 1:** Sonar configurations.

Sonar configuration (Blueview P900-90)	—
Frequency	900 kHz
Bandwidth	2.95 kHz
Field-of-view	90°
Range	10 or 60 m
Beam width	1° × 20°
Beam spacing	0.18°
# of beams	512
# of rays	11 or 114
Source level	220 dB re *μ*Pa

**FIGURE 7:**
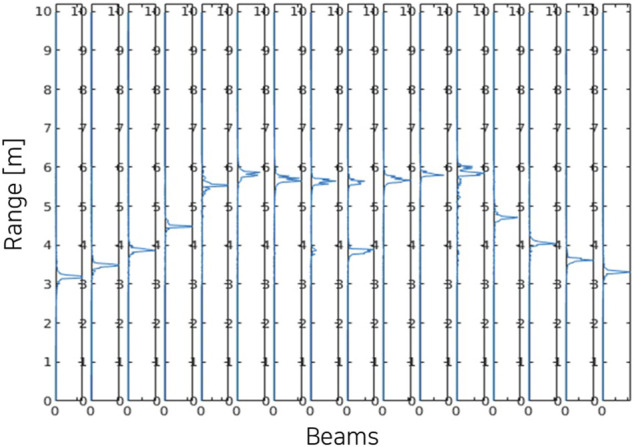
Simulated intensity-range sonar data of a cylinder in a square sonar tank (reflectivity = 1e-3, # of elevation rays = 11).

**FIGURE 8:**
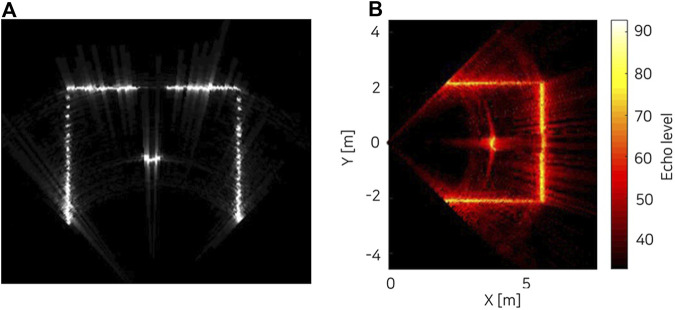
Simulated multibeam echosounder image of a cylinder in a square sonar tank with reflectivity = 1e-3 [**(A)** live-view screenshot in the Gazebo with 11 rays, **(B)** time-averaged image colorized using MATLAB with 114 rays].

The range-intensity sonar data obtained from the simulator is shown in Figure 7 for 16 beams among a total of 512 beams. It shows intensity peaks at the range where the rays hit target objects in the environments. The intensity-range data can be converted into the final sonar image by mapping onto polar coordinates using azimuth and elevation angles of each beam. Figure 8 shows the live-view window in Gazebo (left) and time-averaged image colorized using MATLAB for visualization of the scattering effect (right). In the live-view, the data is manipulated to scale into integer format to match actual sonar data with a controllable gain. Here, both the sonar tank and the target cylinder are set to have 1e-3 reflectivity parameter value.

The result shows the target object and the sonar tank in the final image. Also, the beam scattering of the signal is shown in the vicinity of the target cylinder on the colorized image. Table 2 shows the refresh rate of the sonar image as measured on a workstation with an Intel i9-9900K 3.6 GHz, and a Nvidia GeForce RTX 2080Ti. The most computationally demanding block is the summation that limits maximum range. If the number of rays and the maximum range are reduced, a refresh rate of above 10 Hz is achievable with this hardware, providing real-time sonar data.

**TABLE 2:** The refresh rate and calculation time of the sonar image for various sonar configurations.

		Full calculation	Ray reduced	Ray/Range reduced
Range (m)		60	60	10
# of rays		114	11	11
Refresh rate (Hz)		0.5	3.0	10
Time (s)	Ray signal	0.3	0.02	0.00
	Summation	1.26	0.16	0.04
	Correction	0.05	0.05	0.01
	FFT	0.03	0.03	0.00

**FIGURE 9:**
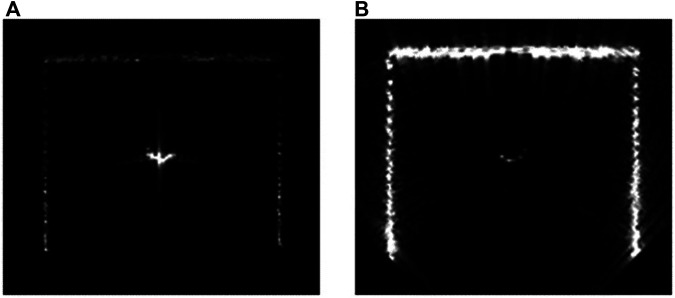
Simulated multibeam echosounder image of various reflectivities [**(A)** low sonar tank reflectivity, **(B)** low target cylinder reflectivity].

It is often the case that the target objects in the environment have different reflectivity according to their material properties. By prescribing the reflectivity parameter for each object model in the scene, the sonar amplitude is calculated accordingly. Figure 9 shows reflectivity value set to 1e-5 for the sonar tank (left) and the target cylinder (right).

**FIGURE 10:**
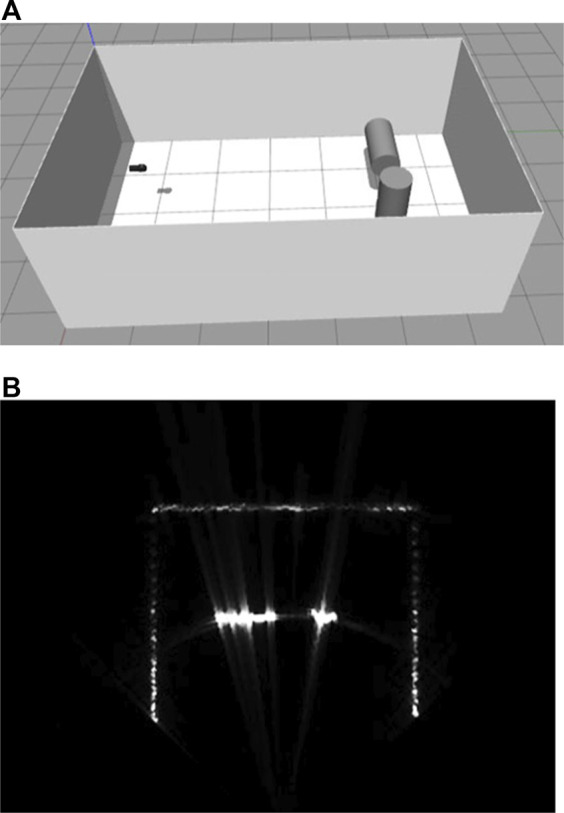
Simulated multibeam echosounder image of two cylinder targets [**(A)** simulation environment, **(B)** live-view sonar image].

**FIGURE 11:**
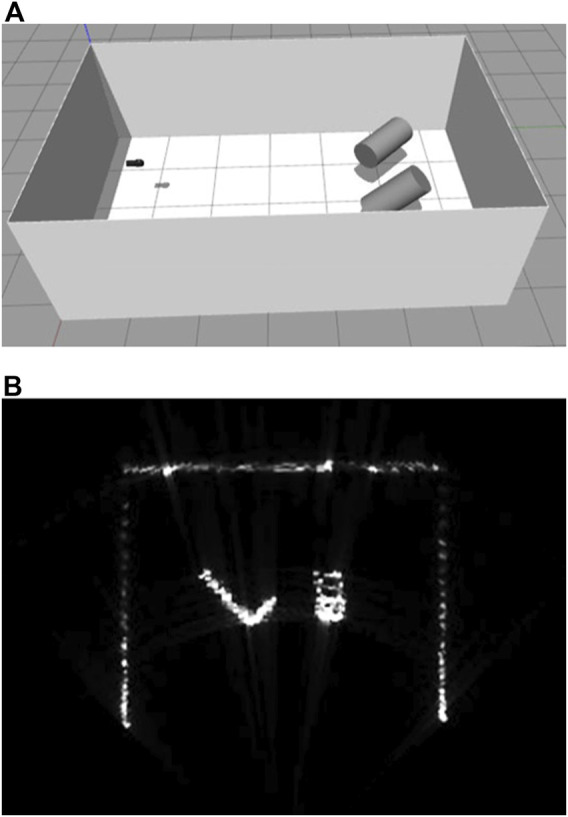
Simulated multibeam echosounder image of two tilted cylinder targets [**(A)** simulation environment, **(B)** live-view sonar image].

Figure 10 shows two cylinders in the sonar tank. Here, the reflectivity of the sonar tank is set to 1e-4 and cylinders to 1e-2. When the two cylinders are tilted as in Figure 11, the amplitude gradient for the difference of distances are shown on the surface of a cylinder on the right and the difference of the incident angles on the left, as well as the blockage effect on the outer wall of the sonar tank.

## 4 Conclusion

In this article we have developed a method to simulate physical-based and high-fidelity multibeam echosounder acoustic perception that underwater manipulation strategies and systems can exploit for development, testing and evaluation. The contributions of this research are the implementation of the point-based scattering model to represent target scattering to produce realistic coherent image speckle and the correct point spread function, as well as the usage of GPU parallelization to obtain real-time refresh rate of up to 10 Hz. A fruitful area of future work would be to implement a more physically based scattering model, such as the Kirchhoff approximation (Abawi, 2016).

The multibeam echosounder developed can provide a sonar image and the underlying physical intensity-range time series signal data approximating what would be produced by an actual sonar. It is implemented as a plugin in the Gazebo framework and released as an open source project for users to manipulate for their uses, such as benchmarking against real sensors for quantitative comparisons. The parameterization of the plugin allows users to simulate various echosounders on the market. Accurate physics-based sensor modeling is a step toward simulating more realistic perception for manipulation, in order to leverage existing manipulation methods in the underwater domain.

## Data Availability

The datasets and source codes in this study are publicly available as open-source and can be found here: https://github.com/Field-Robotics-Lab/nps_uw_multibeam_sonar.

## Footnotes

1 http://www.teledynemarine.com/Lists/Downloads/p900-datasheet-hr.pdf.

2 https://github.com/Field-Robotics-Lab/nps_uw_multibeam_sonar.

3 https://developer.nvidia.com/cuda-toolkit.

4 https://github.com/apl-ocean-engineering/hydrographic_msgs.

## References

[B1] Abawi A. T. (2016). Kirchhoff scattering from non-penetrable targets modeled as an assembly of triangular facets. The J. Acoust. Soc. America 140, 1878–1886. 10.1121/1.4962735 27914371

[B2] Boehme H. Chotiros N. P. Rolleigh L. D. Pitt S. P. Garcia A. L. Goldsberry T. G. (1985). Acoustic backscattering at low grazing angles from the ocean bottom. Part I. Bottom backscattering strength. J. Acoust. Soc. America 77, 962–974. 10.1121/1.392064

[B3] Brown D. C. Johnson S. F. Olson D. R. (2017). A point-based scattering model for the incoherent component of the scattered field. J. Acoust. Soc. Am. 141, EL210–EL215. 10.1121/1.4976584 28372149

[B4] Cerqueira R. Trocoli T. Albiez J. Oliveira L. (2020). A rasterized ray-tracer pipeline for real-time, multi-device sonar simulation. Graphical Models 111, 101086. 10.1016/j.gmod.2020.101086

[B5] Cerqueira R. Trocoli T. Neves G. Joyeux S. Albiez J. Oliveira L. (2017). A novel gpu-based sonar simulator for real-time applications. Comput. Graphics 68, 66–76. 10.1016/j.cag.2017.08.008

[B6] Cieslak P. Ridao P. Giergiel M. (2015). Autonomous underwater panel operation by GIRONA500 UVMS: A practical approach to autonomous underwater manipulation. In *2015* IEEE Int. Conf. Robotics Automation (Icra). 529–536. 10.1109/ICRA.2015.7139230

[B7] Cook D. Vardy A. Lewis R. (2014). “A survey of auv and robot simulators for multi-vehicle operations,” in 2014 IEEE/OES Autonomous Underwater Vehicles (AUV) (New Jersey: IEEE), 1–8. 10.1109/auv.2014.7054411

[B8] DeMarco K. J. West M. E. Howard A. M. (2015). “A computationally-efficient 2D imaging sonar model for underwater robotics simulations in Gazebo,” in OCEANS 2015 - MTS/(Washington: IEEE). 1–7. 10.23919/OCEANS.2015.7404349

[B9] España A. L. Williams K. L. Plotnick D. S. Marston P. L. (2014). Acoustic scattering from a water-filled cylindrical shell: Measurements, modeling, and interpretation. J. Acoust. Soc. America 136, 109–121. 10.1121/1.4881923 24993199

[B10] Goodman J. W. (2015). Statistical Optics. New Jersey: John Wiley & Sons.

[B11] Harris F. J. (1978). On the use of windows for harmonic analysis with the discrete Fourier transform. Proc. IEEE 66, 51–83. 10.1109/proc.1978.10837

[B12] Holland C. (2005). On errors in estimating seabed scattering strength from long-range reverberation. J. Acoust. Soc. America 118, 2787–2790. 10.1121/1.2048947

[B13] Jackson D. R. Richardson M. D. (2007). High-Frequency Seafloor Acoustics. Berlin: Springer.

[B14] Kargl S. G. Espana A. L. Williams K. L. Kennedy J. L. Lopes J. L. (2015). Scattering from Objects at a Water-Sediment Interface: Experiment, High-Speed and High-Fidelity Models, and Physical Insight. IEEE J. Oceanic Eng. 40, 632–642. 10.1109/joe.2014.2356934

[B15] Koenig N. Howard A. (2004). “Design and use paradigms for Gazebo, an open-source multi-robot simulator,” in IROS (Japan: Sendai). 10.1109/IROS.2004.1389727

[B16] Lambert J. H. (1760). Photometria sive de mensure de gratibus luminis, colorum umbrae. Augsburg: Detleffsen for the widow of Eberhard Klett.

[B17] Lewis J. A. (1998). Marine biofouling and its prevention on underwater surfaces. Mater. Forum 22, 41–61.

[B18] Manhães M. M. M. Scherer S. A. Voss M. Douat L. R. Rauschenbach T. (2016). “Uuv simulator: A gazebo-based package for underwater intervention and multi-robot simulation,” in OCEANS 2016 MTS/IEEE Monterey (New Jersey: IEEE), 1–8. 10.1109/oceans.2016.7761080

[B19] Olson D. R. Lyons A. P. Sæbø T. O. (2016). Measurements of high-frequency acoustic scattering from glacially eroded rock outcrops. J. Acoust. Soc. America 139, 1833–1847. 10.1121/1.4945589 27106331

[B20] Palomer A. Ridao P. Youakim D. Ribas D. Forest J. Petillot Y. (2018). 3D laser scanner for underwater manipulation. Sensors 18, 1086. 10.3390/s18041086 PMC5948579 29617303

[B21] Prior M. K. (2005). Experimental investigation of the angular dependence of low-frequency seabed reverberation. IEEE J. Oceanic Eng. 30, 691–699. 10.1109/joe.2005.862093

[B22] Rice S. O. (1944). Mathematical analysis of random noise. Bell Syst. Tech. J. 23, 282–332. 10.1002/j.1538-7305.1944.tb00874.x

[B23] Sanz P. J. Peñalver A. Sales J. Fornas D. Fernández J. J. Pérez J. (2013). Grasper: A multisensory based manipulation system for underwater operations. In *2013* IEEE Int. Conf. Syst. Man, Cybernetics. 4036–4041. 10.1109/SMC.2013.689

[B24] Teledyne BlueView Inc (2015). Teledyne blueview p900 series datasheet. . http://www.teledynemarine.com/Lists/Downloads/p900-datasheet-hr.pdf (Last accessed on 04 30, 2021).

[B25] Tutsoy O. Barkana D. E. (2021). Model free adaptive control of the under-actuated robot manipulator with the chaotic dynamics. *ISA Trans*. 10.1016/j.isatra.2021.02.006 33610316

[B26] Urick R. J. (2013). Principles of Underwater Sound. third edn. Connecticut: Peninsula Publishing.

[B27] UW-APL (2021). hydrographic msgs . https://github.com/apl-ocean-engineering/hydrographic_msgs (Last accessed on 04 30, 2021).

[B28] van Trees H. (1971). Detection, Estimation and Modulation Theory: Part III Radar-Sonar Signal Processing and Detection of Signals in Noise. New Jersey: Wiley.

[B29] Vingelmann P. Fitzek F. H. (2020). Cuda. California: NVIDIA.

[B30] Williams K. L. Kargl S. G. Thorsos E. I. Burnett D. S. Lopes J. L. Zampolli M. (2010). Acoustic scattering from a solid aluminum cylinder in contact with a sand sediment: Measurements, modeling, and interpretation. J. Acoust. Soc. America 127, 3356–3371. 10.1121/1.3419926 20550236

